# Pathoimmunological analyses of fatal E11 infection in premature infants

**DOI:** 10.3389/fcimb.2024.1391824

**Published:** 2024-07-09

**Authors:** Wei Luo, Lixia Wang, Zhengrong Chen, Ming Liu, Yixue Zhao, Yucan Wu, Bing Huang, Ping Wang

**Affiliations:** ^1^ Department of Neonatology, Guangzhou Women and Children’s Medical Center, Guangzhou Medical University, Guangzhou, China; ^2^ College of Pediatrics, Guangzhou Medical University, Guangzhou, China; ^3^ Department of Pathology, Guangzhou Women and Children's Medical Center, Guangzhou Medical University, Guangzhou, China; ^4^ Guangzhou Institute of Pediatrics, Guangzhou Women and Children's Medical Center, Guangzhou Medical University, Guangzhou, China; ^5^ Department of Gastroenterology, Guangdong Provincial Key Laboratory of Gastroenterology, Nanfang Hospital, Southern Medical University, Guangzhou, China

**Keywords:** acute fulminant hepatitis, E11, IFNα, IP10, premature male twins

## Abstract

E11 causes acute fulminant hepatitis in newborns. We investigated the pathological changes of different tissues from premature male twins who died due to E11 infection. The E11 expression level was higher in the liver than in other tissues. IP10 was upregulated in liver tissue in the patient group, and might be regulated by IFNAR and IRF7, whereas IFNα was regulated by IFNAR or IRF5.

## 1 Introduction

Echovirus type 11 (E11) is the most prevalent subtype belonging to the family Picornaviridae, genus Enterovirus. In most cases, echovirus infections follow a benign, self-limiting course, manifesting either asymptomatically or with fever. However, the impact on newborns, particularly premature infants, can often lead to severe complications such as myocarditis, liver failure, coagulation disorders with reduced platelets, and even death (Chuang and Huang, 2019; Zhang et al., 2021). In recent years, sporadic outbreaks of E11 infection have been reported in Europe (Grapin et al., 2023; Piralla et al., 2023), and prior to the prevalence of the coronavirus disease 2019, Asia also witnessed widespread outbreaks (Patel et al., 1985; Ho et al., 2020; Chen et al., 2021; Wang et al., 2023b). Reports of serious consequences in premature infants due to this virus have been on the rise. According to World Health Organization data, the proportion of severe infections in newborns has increased from 6.2% in 2016 to 55% in 2022. Clinical manifestations may include coagulation disorders and liver damage, with critically ill patients developing acute fulminant liver failure. However, the reasons for the severe liver damage caused by E11 in premature infants remain unclear, primarily due to the lack of comparative clinical histopathology.

This study described findings on a pair of premature male twins who suffered from liver damage and succumbed to E11 infection. It offered a comprehensive analysis of the pathological and immunological characteristic differences in liver tissues between infants with E11 infection and control group. By combining clinical biochemical indicators, histopathology, and cytokine immunology, we elucidated the abnormal expression of interferon α (IFNα) and interferon-inducible protein-10 (IP10) in severe cases of E11 infection and discussed potential molecular mechanisms. The aim was to offer new insights into the early diagnosis and treatment of severe liver damage caused by E11 infection in premature infants.

## 2 Materials and methods

### 2.1 Study participants

The cases documented in this study involved a pair of premature male twins admitted to the Neonatal Intensive Care Unit (NICU) of Guangzhou Women and Children’s Medical Center during the 2019 E11 outbreak in China. Throat and rectal swabs were collected for E11 detection using polymerase chain reaction amplification and sequencing methods, confirming the presence of E11 infection. Both infants began exhibiting symptoms on the 7th day of life, with patient 1 succumbing on the 20th day and patient 2 on the 26th day after birth.

### 2.2 Control participants

The liver tissue samples from the control group were obtained from three children with normal liver function who required surgery for choledochal cysts. The lung tissue samples were from a 5-month-old male patient undergoing right upper lobe anterior segmentectomy due to congenital pulmonary malformation (type 2). The specimen was extracted from the surrounding normal lung tissue of the lesion.

### 2.3 Histopathological analysis

Liver, lung, and intestinal tissue samples were obtained through needle aspiration. The liver, lung, and intestinal tissues of control participants were collected during surgery. All tissue samples were fixed with 4% paraformaldehyde, embedded in paraffin, and then sectioned (2–4 mm thickness). Morphological evaluation was performed using hematoxylin and eosin (H&E) staining.

### 2.4 Immunofluorescence staining

Paraffin-embedded sections were deparaffinized and incubated in a blocking buffer (PBS with 5% normal goat serum and 0.3% Triton X-100) for 1 hour. Next anti-human IP-10 (Cat# ab9807; Abcam, Cambridge, UK), was added and incubation continued overnight in a wet chamber at 4°C in the dark. Sections were then washed with PBS, incubated with secondary antibodies for 1 hour at room temperature in the dark, and mounted with VECTASHIELD Antifade Mounting Medium with DAPI (H-1200, Vector Laboratories) for nuclear staining. Immunofluorescent images were acquired with a Leica TCS SP8 Inverted Fluorescence Microscope (Leica Microsystems, Buffalo Grove, IL, USA) using a 10×0.32 dry objective lens and analyzed with Leica X image analysis software.

Paraffin-embedded sections were deparaffinized and incubated in a blocking buffer (PBS with 5% normal goat serum and 0.3% Triton X-100) for 1 hour. They were then stained with Anti-Echovirus Blend Antibody (MAB9670; Sigma) overnight in a wet chamber at 4°C in the dark. Sections were then washed with PBS, incubated with Goat Anti-Mouse IgG-Alexa Fluor 488 (ab150117; Abcam) (1:200) for 1.5 hour at room temperature in the dark, and mounted with VECTASHIELD Antifade Mounting Medium with DAPI (H-1200; Vector Laboratories) for nuclear staining. Immunofluorescent images were acquired using a Leica TCS SP8 Inverted Fluorescence Microscope (Leica Microsystems) using a 20×0.75 dry objective lens. Post-acquisition processing (brightness, opacity, contrast, and color balance) was applied uniformly to all images to accurately reflect the original results. For each section, five areas were selected from 200× magnified images for quantification. The number of Echovirus (> 3 μm in diameter) per square millimeter was counted and analyzed using Leica X image analysis software (Leica, Hamburg, Germany) and ImageJ software (National Institutes of Health, MD, USA).

### 2.5 Multiplexed immunohistochemistry

Multiplexed immunohistochemistry was performed by staining 4-µm-thick formalin-fixed, paraffin-embedded whole tissue sections with standard, primary antibodies sequentially and paired with tyramide signal amplification (TSA) seven-color kit (abs50015-100T; Absinbio;China). Afterward, deparaffinized slides underwent DAPI staining. Specifically, these slides were incubated with anti-IP10 antibody (#bs-1502R; Bioss; China) for 30 min and then treated with anti-rabbit/mouse horseradish peroxidase (HRP)–conjugated secondary antibody (#ARH1001EA; Absinbio; China) for 10 min. Labeling was then performed for 10 min using TSA 520, following the manufacturer’s protocols. The slides were then washed with TBST and transferred to a preheated citrate solution (90°C). Subsequently, they underwent heat treatment in a microwave set to 20% of its maximum power for 15 min. The slides were then cooled in the same solution to room temperature and washed with Tris buffer between steps. The same process was repeated for the following antibodies/fluorescent dyes, in the order of anti-IFNa (#NBP2-75930; Novus; USA), anti-IRF7 (#sc-74472; Santa Cruz;USA), anti-IRF5 (#orb192097; Biorbyt;UK), anti-IRF3 (#11904; CST; USA), anti-IFNRA (#orb673775; Biorbyt; UK), and anti-CD68 (#ab955; Abcam; UK). Subsequently, each slide was treated with two drops of DAPI (FP1490; Thermo Fisher Scientific; USA), washed with distilled water, and manually cover slipped. Following this, the slides were air-dried, and pictures were taken using the Aperio Versa 8 tissue imaging system. The images were analyzed using Indica HALO software.

### 2.6 Statistical analysis

Prism 9.0 (GraphPad) software was used for statistical analysis and cartography. The unpaired *t* test was performed to analyze of two groups. Simple linear regression was used to analyze the correlation between the expression of different cytokines. Statistical significance was defined as P < 0.05.

## 3 Results

### 3.1 Cases and laboratory investigations

The twins included in this study were born in June 2019. Both infants exhibited gastrointestinal symptoms 7 days after birth, and their conditions rapidly deteriorated within 1 day. The twins were transferred from an external neonatal department to the NICU at Guangzhou Women and Children’s Medical Center on day 9 of life because of the severity of their illness. They were diagnosed with E11 infection on the 11th day. The twins were also tested for coxsackievirus, Epstein-Barr virus, herpes simplex virus, cytomegalovirus, human parvovirus, and adenovirus after admission, all of which were negative.

#### 3.1.1 Birth history

The twins were conceived through *in vitro* fertilization, specifically as monochorionic diamniotic. Their gestation age were 32 + ^5^ weeks. The firstborn (P1) experienced premature rupture of membranes for 25 hours, with clear amniotic fluid and umbilical cord wrapped around the neck once. No complications were observed during delivery, and both infants were admitted to the local neonatal department due to prematurity and low birth weight. P1 and P2 had birth weights of 1.65 kg (22nd percentile) and 1.8 kg (34th percentile), respectively. The mother had regular prenatal check-ups, with no symptoms of vomiting, diarrhea, or fever during pregnancy. No instances of gestational diabetes or gestational hypertension were observed. Apart from the administration of dexamethasone for fetal lung maturation, no other medications were administered before delivery.

#### 3.1.2 Clinical course of P1

P1 started vomiting frequently on the 7th day after birth and developed uncorrectable hypotension, oliguria, generalized edema, skin ecchymosis (particularly in the limbs), and hypoxemia by the 8th day. The laboratory results revealed metabolic acidosis, significant coagulation abnormalities, and abnormal liver function. On the 9th day after birth, P1 was transferred to the NICU at Guangzhou Women and Children’s Medical Center. On admission, P1 exhibited poor responsiveness, irregular breathing, pronounced edema, anuria, and laboratory findings revealed respiratory and metabolic acidosis, severe anemia, thrombocytopenia, coagulation disorders, and abnormal liver function. Despite interventions, including mechanical ventilation, antimicrobial therapy, blood product transfusions, and various vasopressors, P1’s condition continued to deteriorate. Continuous renal replacement therapy(CRRT) was initiated on the 11th day, with plasma exchange performed on the 17th and 19th days. Complications, such as pulmonary arterial hypertension, occurred, and the family chose to discontinue treatment on the 20th day due to multi-organ bleeding.

#### 3.1.3 Clinical course of P2

P2 also experienced apnea on the 7th day after birth, accompanied by a decrease in oxygen saturation. On the same day, P2 began vomiting coffee-ground-vomited. On the 8th day, P2 exhibited the same symptoms as P1, and was also transferred to the same hospital. Upon admission, P2 displayed poor responsiveness, irregular breathing, significant edema, oliguria, and laboratory findings revealed with respiratory and metabolic acidosis, severe anemia, thrombocytopenia, coagulation disorders, and liver dysfunction. Like P1, P2 underwent various treatments, including respiratory support, inhaled nitric oxide, antimicrobial agents, immunoglobulin, blood product transfusions, vasopressors, and fluid resuscitation. CRRT was initiated on the 13th day, with four rounds of plasma exchange performed. Despite active treatment, P2’s clinical symptoms persisted, accompanied by multi-organ damage, severe acidosis, hypotension, and hypoxemia. Unfortunately, resuscitation efforts were unsuccessful, and P2 succumbed on the 26th day after birth.

The male premature twins infected with E11 exhibited a swift disease progression and severe symptoms. Gastrointestinal symptoms such as vomiting were the first symptoms, and the disease rapidly developed into multiple organ failure within a day, with particularly severe liver damage. This was evident in the form of fulminant hepatic failure characterized by a rapid increase in liver enzymes and bilirubin levels, along with abnormalities in coagulation factors (**Table 1**).

**Table 1:** Laboratory result about a pair of premature male twins with E11 infection.

Laboratory findings	P1	P2	Reference range
Minimum platelet value (10^9/L)	9.0	7.0	140-440
Minimum hemoglobin value (g/L)	46.0	60.0	135-195
ALT peak (U/L)	228.0	285.0	9-50
AST peak (U/L)	3055.0	3255.0	5-60
TBIL peak (umol/L)	274.0	686.7	2-17
DBIL peak (umol/L)	36.3	132.0	2-13.7
γ-GT peak (U/L)	206.0	145.0	10-60
Minimum albumin (g/L)	20.8	24.8	40-50
APTT (s),mean (min,max)	112.5 (46.8-180.0)	91.8 (48.1-180,0)	28-45
PT (s),mean (min,max)	26.0 (14.2-120.0)	27.8 (15.3-98.1)	11-15
TT (s),mean (min,max)	98.5 (19.5-240.0)	78.9 (21.3-240.0)	14-21
FIB (g/L),mean (min,max)	2.2 (0.6-3.4)	2.1 (0.6-5.2)	2-4g/L
IL6 (pg/ml),mean (min,max)	113519.5 (4760.0-162890.2)	19921.3 (1760.66-53500.4)	0-8.88
IL8 (pg/ml),mean (min,max)	112353.4 (3074.3-1703.0)	58304.3 (1313.67-170306.6)	0-15.71
IL10 (pg/ml),mean (min,max)	381.6 (28.1-609.0)	308.1 (5.65-1303.7)	0-8.14
IFNα (pg/ml),mean (min,max)	450.0 (328.5-775.1)	201.1 (44.4-656.8)	–
IP10 (pg/ml),mean (min,max)	126512.0 (62617.2-135872.7)	89320.7 (62224.6-120162.1)	–

### 3.2 Histopathological analysis of the liver, intestine, and lungs

**Figure 1:**
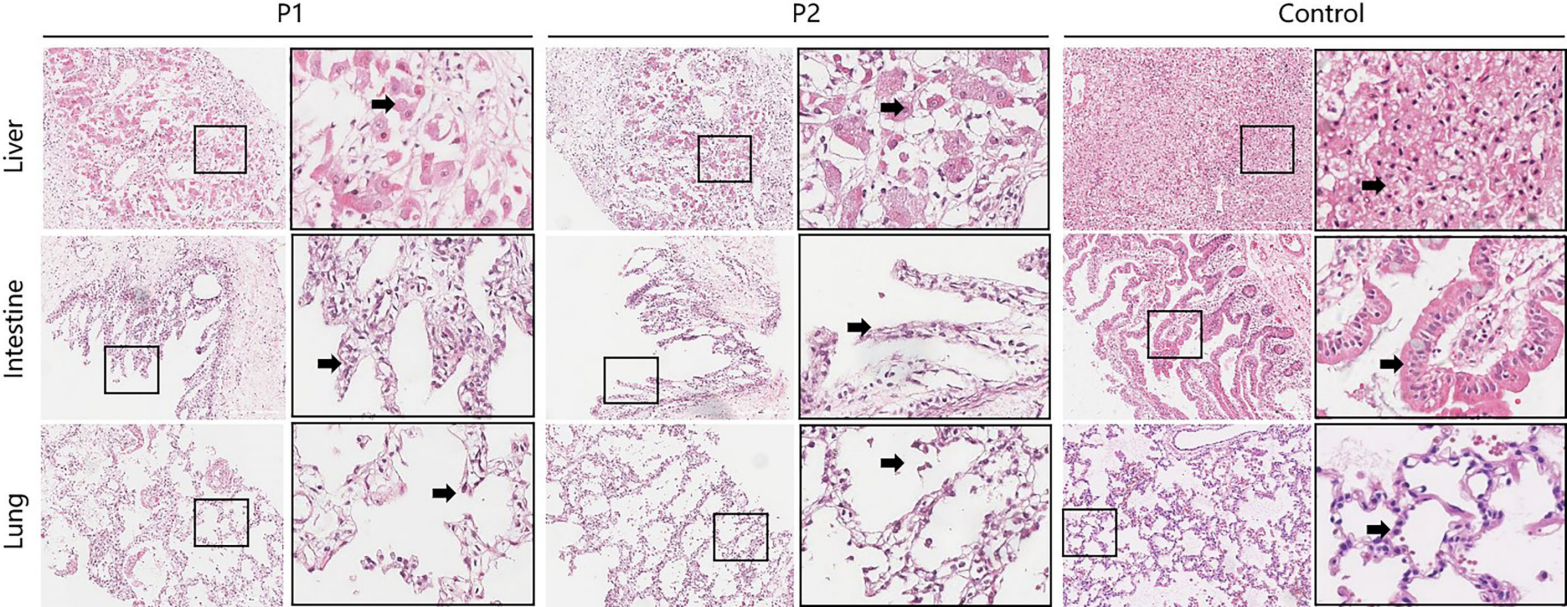
Pathological examination of multiple organ tissues between the deceased twins and the control group. (HE stain; 20×10). The column on the right is ×4 magnified on the left. Liver sections of twins showed that multifocal patchy necrosis of hepatocytes, fragmentation, and dissolution of hepatocyte nuclei(arrow), accompanied by a significant infiltration of inflammatory cells, primarily lymphocytes, and marked expansion of the portal areas. The liver cells of control group did not show degeneration or accumulation(arrow), and the portal areas were slightly enlarged but structurally intact, with minimal lymphocyte infiltration. Intestine sections of twins showed the intestinal villi were absent and the intestinal epithelial cells were reduced and disorganized (arrow). The control group revealed dense villi in the mucosal layer of the small intestine, well-arranged and tightly packed intestinal epithelial cells with a clear brush border (arrow), and clusters of lymphocytes in the lamina propria. Lung sections of twins exhibited inflammatory cell infiltration in the interstitium of the lungs, accompanied by pulmonary emphysema(arrow). Lung sections of the control showed the structure of bronchioles and alveoli is intact.

In the liver tissue of the twins, the hepatic lobular structure was not clearly visible under H&E staining. Multifocal patchy necrosis of hepatocytes, fragmentation, and dissolution of hepatocyte nuclei were observed. This was accompanied by a significant infiltration of inflammatory cells, primarily lymphocytes, and marked expansion of the portal areas. In contrast, the liver tissue from the control group exhibited a clear hepatic lobular structure at low magnification. The hepatocytes did not show degeneration or accumulation, and the portal areas were slightly enlarged but structurally intact, with minimal lymphocyte infiltration. Compared with the control, the intestinal villi were absent and the intestinal epithelial cells were reduced and disorganized in the twins. The microscopic examination of lung tissue from the control group revealed intact structures of small bronchi and alveoli. In contrast, the deceased twins exhibited inflammatory cell infiltration in the interstitium of the lungs, accompanied by pulmonary emphysema (**Figure 1**).

### 3.3 Expression of E11 and IP10 in different tissues

**Figure 2:**
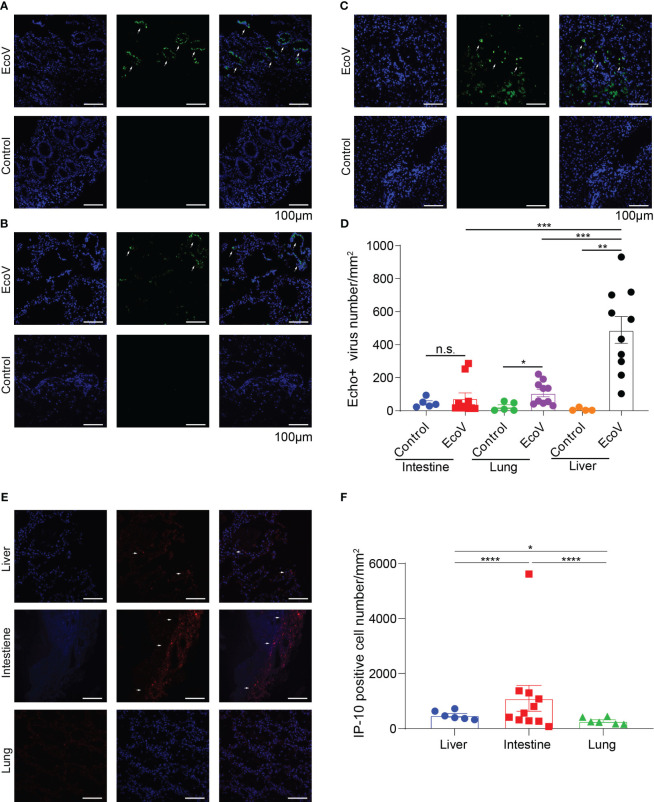
**(A-C)** show the expression and distribution of representative immunofluorescence of E11 in the intestine, lungs, and liver of the deceased twins and control group. Green represents E11, and blue represents the nucleus. White arrows indicate E11. **(D)** displayed statistical analysis based on immunofluorescence in **(A-C, E)** show the expression and distribution of IP10 in the different tissues. Red represents IP10, and blue represents the nucleus. White arrows indicate IP10. **(F)** displayed statistical analysis based on the observed immunofluorescence in **(E)**. N.S, no significace, * P value <0.05, ** P value <0.01, *** P value <0.001 and **** P value <0.001.

To further investigate the distribution of E11 in different tissues and organs, we used immunofluorescence staining to compare the expression and distribution of E11 in the lungs, intestines, and liver of twins. The results showed that E11 was expressed in the lungs, intestines, and liver, with the highest number of virus positive cells in the liver. In addition, E11 exhibited focal aggregation in intestinal epithelial cells rather than a diffuse distribution. IP10 expression to be expressed in different tissues in the intestine, lungs, and liver, with the highest expression observed in the intestine, followed by the liver, and rarely expressed in the lungs. In summary, E11 mainly localized to the liver, followed by the intestine and lungs, and its distribution might correlated positively with IP10 expression (**Figure 2**).

### 3.4 Expression of IFNα upstream factors in liver tissue

Our previous studies reported decreased peripheral-blood levels of IFNα in infants with severe liver damage compared with those with mild symptoms without liver dysfunction (Wang et al., 2023b). We conducted multicolor immunofluorescence staining to analyze the expression of IFNα, interferon α receptor(IFNAR), IP10, IRF3, IRF5, and IRF7 in the liver tissue of the twins and compared it with that in the control group. We selected IFNα, IFNAR, and IP10 as Panel 1, and IRF3, IRF5, and IRF7 as Panel 2.

**Figure 3:**
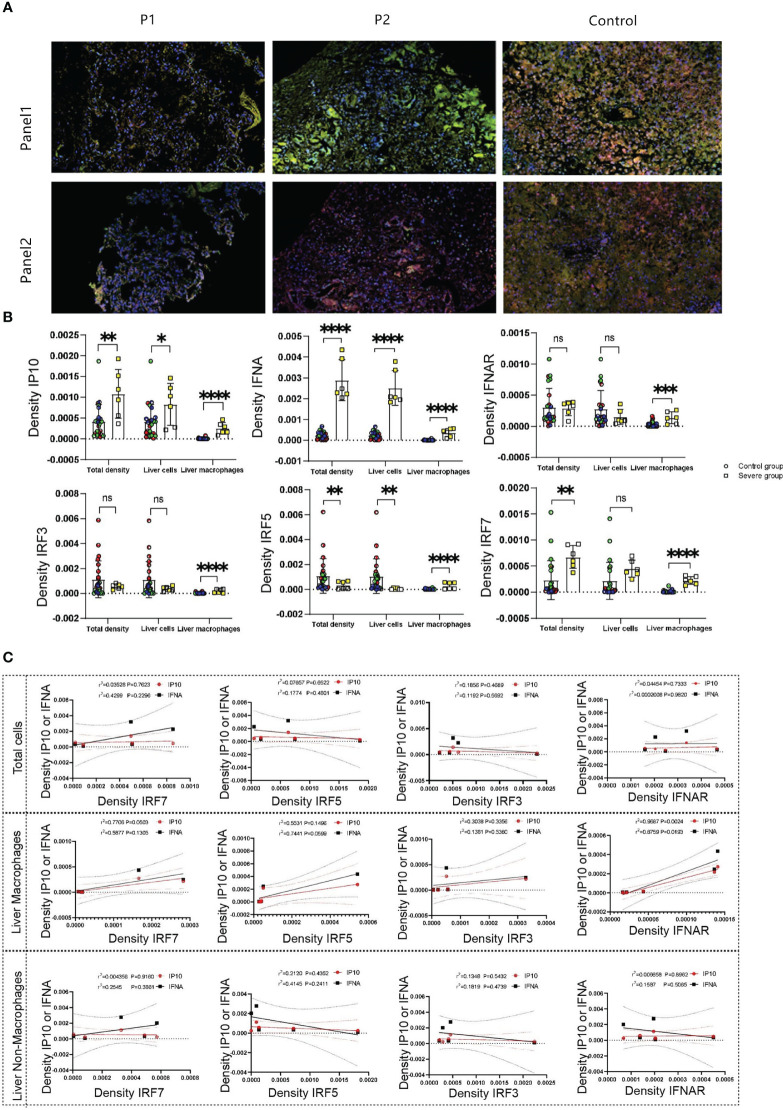
**(A)** show the expression and distribution of representative multicolored immunofluorescence of IFNα, IFNRA, IP10, IRF3, IRF5, and IRF7 in liver tissue of the deceased twin and control groups. IFNα, IFNAR, and IP10 as Panel 1(green represents IP10, yellow represents IFNα, orange represents IFNRA, pink represents CD68, and blue represents the nucleus), and IRF3, IRF5, and IRF7 as Panel 2(green represents IRF7, yellow represents IRF5, orange represents IRF3, pink represents CD68 and blue represents the nucleus). **(B)** displayed statistical analysis based on **(A)**. **(C)** displayed IP10 and IFNα correlation with IRF7, IRF5, IRF3, and IFNAR, respectively. N.S, no significace, * P value <0.05, ** P value <0.01, *** P value <0.001 and **** P value <0.001.

The results showed that both hepatocytes and Kupffer cells exhibited a significant increase in IP10 expression in severe cases, consistent with our peripheral blood serum results. However, elevated IFNα levels were observed only in Kupffer cells, not in hepatocytes (**Figures 3A, B**; **Supplementary Figure 1**). Additionally, IFNAR expression was higher in Kupffer cells of severe cases compared with the control group, whereas no statistical difference was observed in hepatocytes (**Figure 3B**). We found that the levels of IRF3, IRF5, and IRF7 in Kupffer cells were higher in severe cases compared with the control group. However, IRF5 expression in hepatocytes decreased in severe cases (**Figure 3B**).

We performed multicolor immunofluorescence-based correlation analysis between IP10, IFNα, and the transcription factors IRF3, IRF5, and IRF7 to identify the transcription factors regulating IP10 and IFNα. The results indicated no significant correlation between these inflammatory factors and IRF3, IRF5, or IRF7 at the total cellular level (**Figure 3C**). However, in liver Kupffer cells, a weak positive correlation was observed between IRF7 and both IP10 and IFNα. In contrast, in non-Kupffer cells within the liver, only IRF7 showed a significant positive correlation with IP10 (**Figure 3C**). Notably, in liver Kupffer cells, both IP10 and IFNα exhibited a significant positive correlation with IFNAR. In summary, the results suggested that IP10 from liver Kupffer cells might be regulated by both IFNAR and IRF7, whereas IFNα was primarily regulated by IFNAR or IRF5.

## 4 Discussion

E11 is notably associated with sepsis and acute fulminant hepatitis in newborns, often leading to neonatal deaths (Bubba et al., 2017; Zhang et al., 2021). Severe cases have received increased attention from researchers. Reports from different countries in recent years have documented cases of severe liver failure and even death in newborns infected with E11 (Grapin et al., 2023; Piralla et al., 2023), with premature birth and male sex identified as potential susceptibility factors, but the specific pathogenic mechanisms remain unclear. One study reported nine cases, with eight being male premature twins (Grapin et al., 2023). The cases presented in this study also concern male premature twins infected around the age of 1 week, rapidly progressing to acute fulminant hepatitis, similar to cases reported in Italy and France (Grapin et al., 2023; Piralla et al., 2023). Based on clinical manifestations, this study delved deeper into the pathogenesis from the perspectives of histopathology and cytokine immunology.

This study was novel in comparing the histopathological changes in different tissues following E11 infection with normal controls. Viral quantification studies were also conducted on various tissue samples from patients. Unlike other enteroviruses such as *Enterovirus A71* (EV71) (Huang et al., 2020; Moshiri et al., 2023) and *Coxsackievirus B3* (CVB3) (Massilamany et al., 2014; Garmaroudi et al., 2015), severe E11 infection prominently manifests as notable liver damage. The histopathological findings from liver autopsy samples of the twins were consistent with the pathological changes of liver failure. Immunofluorescence staining revealed a significantly higher number of virus-positive cells in the liver compared with the intestine and lungs. This confirmed, for the first time in human samples, that the liver was a crucial target organ for E11-induced sepsis. A previous study by Wells et al. reported a similar phenomenon in mouse model of echovirus infection (Wells et al., 2022), but severe E11 infection occurred only when the IFNAR gene was knocked out. However, in this study, a comparison of IFNAR expression in liver tissue between the case and control groups revealed that the expression in Kupffer cells of the case group was significantly higher than that in the control group, with no statistical difference in hepatocytes. This result suggested that, although the key point leading to severe infection in animal experiments was the knockout of IFNAR, the key regulatory mechanism in severe cases might be associated with abnormalities in other key points of the IFNα signaling pathway.

IFNα originating from extraepithelial sources is crucial for constraining enterovirus replication in the human intestine and is secreted by most types of cells (Vilcek and Oliveira, 1994). They can initiate signal transduction through autocrine or paracrine mechanisms, forming ternary receptor complexes with their respective receptors to induce IFN-mediated immune responses (Schneider et al., 2014; Saxena et al., 2017).Our team previously validated significantly lower levels of IFNα in the peripheral-blood serum of patients with severe liver injury infected with E11 compared with mild cases (Wang et al., 2023b). Animal experiments have demonstrated that, without knocking out the IFNAR gene, E11 infection remains localized to the intestine. However, after the gene is knocked out, blocking the normal function of IFNα allows E11 infection to spread systemically (Wells et al., 2022). The decreased levels of IFNα in patients with severe liver injury infected with E11 might contribute to the virus spreading and causing liver failure in severe cases. The expression of upstream transcription factors of IFNα in the liver tissue of these twins was examined to further explore the specific regulatory mechanisms. The expression level of IRF5 in the liver tissue was observed to be significantly lower than that in the control group. A weak positive correlation between IFNα and IRF5 was observed in Kupffer cells within the liver. Numerous studies have demonstrated that IRF3, IRF5, and IRF7 are involved in the activation and secretion of Type-I IFNs after viral invasion, playing a synergistic role in this process (Barnes et al., 2001; Barnes et al., 2004; Paun et al., 2008; Jefferies, 2019; Nie et al., 2019). Upon activation of Toll-like receptors (TLRs) 7, 8, or 9 by exogenous nucleic acids, a supramolecular organizing center is formed, connecting endosomal TLRs with enzymes and ubiquitin-modified proteins. The formation of this complex in the early endosome leads to the activation of NF-κB and the transcription of pro-inflammatory cytokines. Simultaneously, IRF5 is recruited to lysosomes and undergoes phosphorylation. Subsequently, IRF5 translocates to the cell nucleus, initiating the transcription of Type-I IFN genes (Elkon and Briggs, 2020). IRF5 may directly impact the secretion of IFNα and also influence the synergistic action among various regulatory factors, affecting the body’s antiviral capabilities. Therefore, IRF5 may be one of the therapeutic targets for treating acute fulminant liver failure caused by E11 in newborns. Additionally, studies have reported that viral proteins selectively act on a specific target within the IFN signaling pathway, causing damage to the IFN signal transduction pathway (Rasti et al., 2019; Ji et al., 2023). Further studies are needed to confirm whether the downregulation of IRF5 expression in severe E11 infection is due to a specific viral component.

IFNα and IP10 are two key factors that modulate the immune reaction of type 1 T helper cells (Th1 cells). IP10, a member of the CXC chemokine subfamily, acts as a chemoattractant for activated T cells (Chen et al., 2013). It increases the migration of Th1 cells. IFNα prompts the proliferation of cytotoxic CD8^+^ T cells and the release of IFNγ (Hervas-Stubbs et al., 2010). The dysfunction of excreting IFNα in premature infants cannot stimulate the anti-E11 reaction. More IP10 was produced to recruit additional lymphocytes as feedback to the low level of IFNα (Brownell and Polyak, 2013; Dai et al., 2020; Yu et al., 2022; Zhang et al., 2022). The elevated expression of IP10 in the liver of the twins in this study aligned with previous findings associating IP10 levels with hepatic injury. *In vitro* experiments have demonstrated that E11 can induce IP10 expression (Drummond et al., 2017), a phenomenon not observed with other enteroviruses such as EV71 and CVB3. The elevated expression of IP10 appears to be a characteristic feature of E11 among enteroviruses. Animal studies have further confirmed that E11 induces the secretion and aggregation of IP10 in the liver, leading to severe hepatic damage (Wells et al., 2021). Following invasion of liver cells, E11 triggers significant IP10 secretion, attracting macrophages and natural killer cells to the liver. This leads to acute inflammatory responses and cytotoxic immune reactions, ultimately resulting in extensive hepatocyte necrosis. Therefore, inhibiting IP10 may serve as one of the therapeutic targets for treating E11-induced liver injury. Animal experiments have revealed that, during sepsis, myeloid-related protein 8/14 (MRP8/14) induces sustained IP10 production through the IFN-β-JAK1/TYK2-STAT1-IRF7 pathway (Wang et al., 2023a). Our findings were consistent with the results of these animal experiments, suggesting that IRF7 might also be one of the targets implicated in severe E11 infection leading to liver injury.

E11, similar to other enteroviruses, primarily spreads through the fecal–oral route, using the gastrointestinal tract as its main entry point (Morosky et al., 2019). Virus-positive cells were detected in the intestinal epithelium of the twins examined in this study, exhibiting a focal rather than diffuse distribution. This pattern might be linked to the involvement of various cell types in the viral invasion of the intestinal epithelium. Previous studies have indicated E11 replication in intestinal epithelial cells and enteroendocrine cells, but not in goblet cells (Drummond et al., 2017). Our observations also revealed intestinal damage in severe cases, characterized by the disappearance of intestinal villi structure and the presence of reduced and disorganized intestinal epithelial cells. This finding contrasted with the results of animal experiments where virus replication was confined to intestinal epithelial cells, and no damage to the intestinal epithelium was observed (Wells et al., 2022).

This study was the initial investigation into immunological characteristics using patient tissue samples, validating findings from animal experiments and proposing potential mechanisms for developing severe illness. However, its limitations included the absence of liver tissue samples from patients with mild E11 infection for comparative analysis, as the control cases involved non-E11-infected individuals. Additionally, this study provided only a preliminary exploration of upstream factors affecting IFNα and IP10, necessitating further clinical data and animal experiments to reveal the regulatory roles of IRF5, IRF7, and IFNAR in severe E11 infection in neonates.

In summary, this study was novel in exploring severe E11 infection in premature male twins at the tissue level. The findings, coupled with multicolor immunofluorescence, might offer initial insights into the potential mechanisms and prospective targets for preventing and treating severe preterm E11 infection.

## Data availability statement

The original contributions presented in the study are included in the article/**Supplementary Material**, further inquiries can be directed to the corresponding authors.

## Ethics statement

The studies involving humans were approved by the Institutional Review Board of Guangzhou Women and Children’s Medical Centre (GWCMC-(2020)63201). The studies were conducted in accordance with the local legislation and institutional requirements. Written informed consent for participation in this study was provided by the participants’ legal guardians/next of kin.

## Author contributions

WL: Data curation, Investigation, Writing – original draft, Writing – review & editing. LW: Formal analysis, Investigation, Software, Writing – original draft. ZC: Project administration, Writing – review & editing. ML: Methodology, Writing – review & editing. YZ: Investigation, Writing – review & editing. YW: Investigation, Writing – review & editing. BH: Data curation, Investigation, Writing – original draft, Writing – review & editing, Formal analysis, Project administration, Software. PW: Data curation, Investigation, Writing – original draft, Writing – review & editing, Methodology, Supervision.

## Supplementary material

Supplementary Figure 1 **(A)** is the Panel 1. It shows the expression and distribution of IP10, IFNα, and IFNRA in liver tissues of the deceased twins and control group, respectively (green represents IP10, yellow represents IFNα, orange represents IFNRA, pink represents CD68, and blue represents the nucleus). **(B)** is the Panel 2. It shows the expression and distribution of IRF3, IRF5, and IRF7 in liver tissues of the deceased twins and control group respectively (green represents IRF7, yellow represents IRF5, orange represents IRF3, pink represents CD68 and blue represents the nucleus).
