# Supplementary figures and images for: Pathoimmunological analyses of fatal E11 infection in premature infants
Source: Front Cell Infect Microbiol. 2024 Jul 9;14:1391824. doi: 10.3389/fcimb.2024.1391824 (PMC11263194; doi:10.3389/fcimb.2024.1391824)

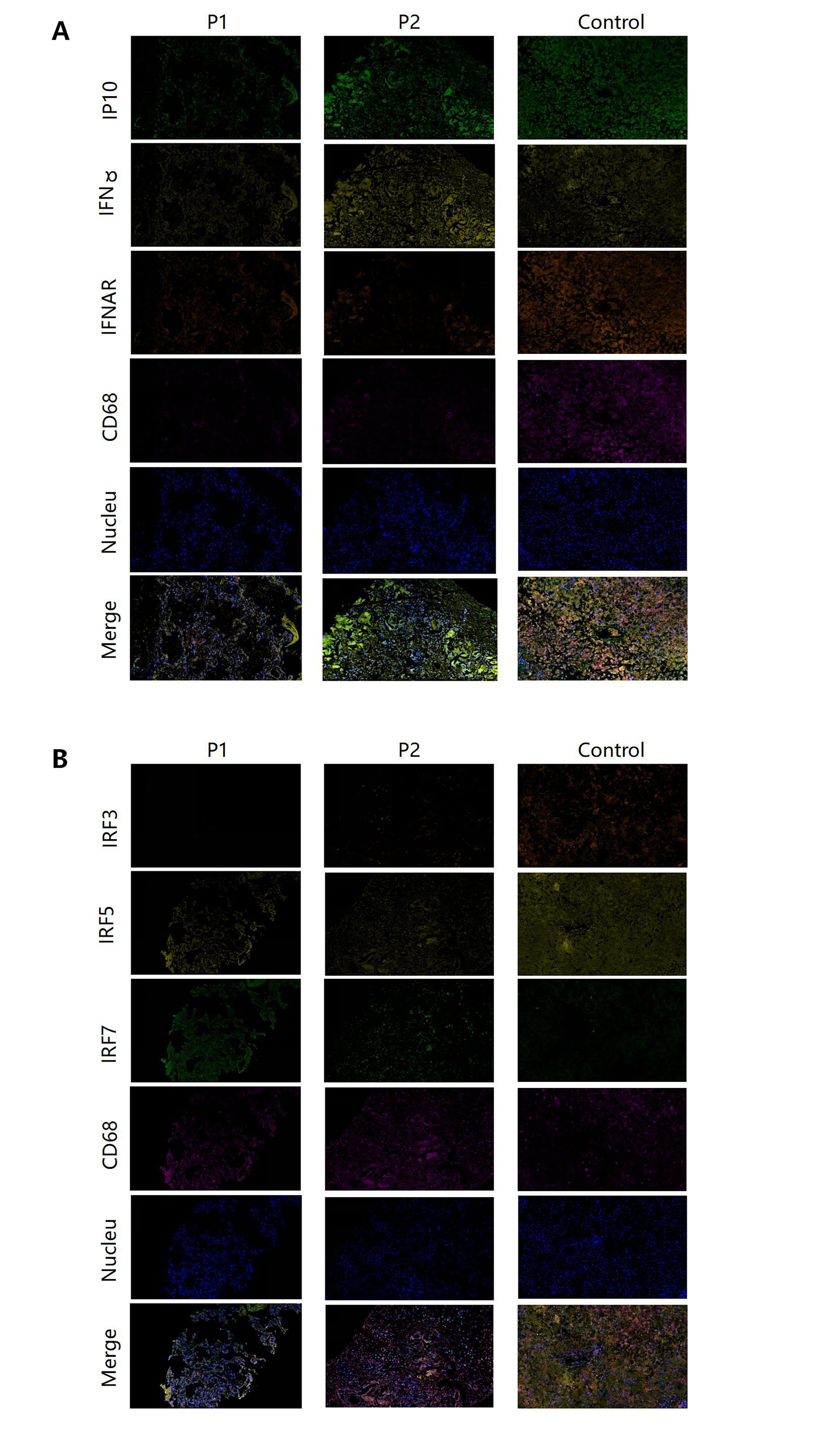

Supplement: Supplementary Figure 1 — (A) is the Panel 1. It shows the expression and distribution of IP10, IFNα, and IFNRA in liver tissues of the deceased twins and control group, respectively (green represents IP10, yellow represents IFNα, orange represents IFNRA, pink represents CD68, and blue represents the nucleus). (B) is the Panel 2. It shows the expression and distribution of IRF3, IRF5, and IRF7 in liver tissues of the deceased twins and control group respectively (green represents IRF7, yellow represents IRF5, orange represents IRF3, pink represents CD68 and blue represents the nucleus). [file Image_1.jpeg]
